# How Human Activity Has Changed the Regional Habitat Quality in an Eco-Economic Zone: Evidence from Poyang Lake Eco-Economic Zone, China

**DOI:** 10.3390/ijerph17176253

**Published:** 2020-08-27

**Authors:** Tianzhu Zhang, Yang Gao, Chao Li, Zhen Xie, Yuyang Chang, Bailin Zhang

**Affiliations:** 1 College of Land Science and Technology, China Agricultural University, Beijing 100193, China; ztz@cau.edu.cn (T.Z.); xiezhen@cau.edu.cn (Z.X.); chang_young@foxmail.com (Y.C.); 2 Key Laboratory of Agricultural Land Quality, Ministry of Natural Resources, Beijing 100193, China; 3 Land Consolidation and Rehabilitation Center, Ministry of Natural Resources, Beijing 100035, China; lichaonongda@163.com; 4 School of Economics and Management, Tianjin Polytechnic University, Tianjin 300387, China; zhangbailin135@163.com

**Keywords:** habitat quality, land-use change, China, eco-economic zone

## Abstract

Human activities such as deforestation and urbanization have affected the regional habitat quality of the Poyang Lake area. To evaluate the evolution of habitat quality and its influencing factors in the area, we used Classification and Regression Trees (CART) to interpret the land-use status and used the InVEST (Integrated Valuation of Ecosystem Services and Tradeoffs) model to analyze the characteristics of changes in habitat quality in the Poyang Lake Eco-Economic Zone (PLEEZ) from 1988 to 2018. The results show that, from 1988 to 2018, land use in the PLEEZ underwent significant changes. The changes in land use led to a gradual increase in habitat degradation and a gradual decrease in habitat quality in the study area. Rapid urbanization notably decreased the habitat quality in the study area. However, at the same time, the ecological protection projects such as returning farmland to forests slowed the decline in habitat quality. Driven by these two factors, habitat quality in the PLEEZ gradually declined but the rate of its decline was suppressed. The findings of this study are of great significance for the coordinated development of social, economic, and ecological development in the PLEEZ and similar areas.

## 1. Introduction

The eco-economic zone is a new regional development model proposed to achieve coordination and balance between social economy and ecology. At present, low carbon and ecology have become central themes and how to build a benchmark eco-economic zone has become a focus of social concern [1]. Some eco-economic zones have been established in China, including Dongting Lake Eco-Economic Zone [2], Poyang Lake Eco-Economic Zone [3], Yellow River Delta Eco-Economic Zone [4], Western Sichuan Plateau Eco-Economic Zone [5], etc. The purpose of establishing an eco-economic zone is to protect the ecology and to develop the economy. However, the rapid development of urbanization has caused cultivated land and high-rise buildings to invade the habitats on which living creatures depend, which poses a huge challenge in protecting biodiversity [6,7,8]. Data from the World Urbanization Prospects: The 2018 Revision report released by the United Nations Department of Economic and Social Affairs shows that 55% of the world’s population lived in cities in 2018, and this percentage is estimated to increase to 68% by 2050. Urbanization is a serious threat to biodiversity. Researchers have determined that the world is experiencing the sixth human-induced mass extinction event [9]. Human behavior fundamentally and irreversibly changes the diversity of life on Earth [10]. The rate of extinction is increasing, and the number of threatened species continues to increase [11]. China is one of the most biodiverse countries in the world and has four global biodiversity hot spots as identified by Conservation International [12]. After the reform and opening up in 1978, China’s urbanization accelerated, and the urbanization rate increased significantly from 17.92% in 1978 to 59.58% in 2018. However, this also seriously threatened ecological security. The latest “China Biodiversity Red List” shows that approximately 22% of vertebrates and approximately 11% of higher plants have gone extinct or are threatened [13]. Due to the increased protection of biodiversity in recent years, many regions have established strictly regulated nature reserves [14,15]. However, due to changes in the global environment, disturbance from invasive species, and human activities, the loss of biodiversity is still a huge challenge facing the world [16,17,18]. Therefore, the rapid and effective assessment of habitat quality is important for regional ecological protection and restoration.

Habitat quality is an indicator that describes the ability of the ecological environment to provide living conditions for living beings and can reflect the status of regional biodiversity [19,20]. It determines the fitness of biological habitats and plays a major role in biodiversity conservation [21,22]. In recent years, habitat quality has gradually become a research hotspot in the field of ecology [23,24,25]. In recent years, habitat research has focused on two perspectives. One is to evaluate the impact of the external environment or human activity on the habitat quality of individual species [26,27,28,29]. These studies first clarify the distribution range and characteristics of the target species and then analyze the threat factors that affect the habitat quality of the target species [29]. These studies have shown that human activities, natural disasters, and species invasions are the main factors affecting the quality of biotic habitats. For example, Giacomazzo et al. studied changes in the quality of fish habitats in the Lake Saint-Pierre basin and showed that runoff from intensively cultivated land leads to deterioration of water quality and changes in aquatic vegetation abundance. Restoring aquatic vegetation and improving water quality are the fundamental ways to promote fish stock recovery [26]. Second, these studies conducted regional habitat quality assessment and its influencing factors, such as the impact of urban expansion on habitat quality and the impact of human activities [30,31], or land-use changes on habitats in nature reserves [32,33]. Similar studies have shown that humans can degrade regional habitat quality [32]. Urban expansion leads to isolation and fragmentation of landscape patterns, destruction of ecosystem integrity, and serious impacts on regional habitat quality [24,34]; the vegetation degradation caused by overgrazing also worsens habitat quality [35]. For instance, Bai et al. analyzed the spatial and temporal characteristics of the landscape pattern and habitat quality in Changchun City using spatial analysis and ecological models based on land-use data. The study shows that urbanization development and construction will seriously threaten the regional habitat quality [30]. The research of Hamilton et al. in Australia shows that population growth and agricultural and mining development have severely degraded the local vegetation and threatened habitat quality [35]. From the perspective of research methods, at this stage, scholars are using models more often to study habitat quality. For example, the EQI (Eco-environmental Quality Index) model [36], InVEST [30,31,32,33], and other models are used for regional habitat quality evaluation. The InVEST model has the advantages of relatively low data requirements and highly visible calculation results. It has been widely used in the study of habitat quality.

Poyang Lake is China’s largest freshwater lake and is located on the south bank of the middle and lower reaches of the Yangtze River in northern Jiangxi Province. It is the only lake of China’s four largest freshwater lakes that is not eutrophic. It is also an important global wetland and the largest migratory bird habitat in Asia. It has gained extensive international attention and influence. The Poyang Lake Basin is known as a paradise for migratory birds in China [37,38]. There are more than 4000 plants and 900 animals in the basin, of which more than 100 are protected by the state; therefore, the basin is of great significance to the natural protection of the middle and lower reaches of the Yangtze River [39]. The study of habitat quality in the Poyang Lake Basin mainly assesses its habitat suitability for individual species. Tang et al. studied how land-use changes in the Poyang Lake area affected the habitat suitability for wintering Anseriformes and pointed out that land-use changes are the principal way to improve the quality of the habitat of wintering Anseriformes [38]. Sun et al. evaluated the quality and spatial distribution characteristics of migratory bird habitats under different land-use scenarios in typical villages in the Poyang Lake region in the future. The study showed that human activities are the main reason for the decline in the quality of migratory bird habitats [40]. However, we found that there is a lack of long-term sequence studies on the evolution of overall habitat quality in the Poyang Lake area. In 2008, the Poyang Lake Eco-Economic Zone (PLEEZ) was established, and the land-use and land cover of the area have undergone major changes [38,41]. Land-use change causes many serious ecological problems and threatens the sustainable development of human society and the continuous supply of ecosystem services [42]. As a basis for sustainable economic development and rational resource use, it is necessary to assess changes in habitat quality in the region.

In addition, accurate land-use/cover data are important for assessing changes in the spatial patterns of regional habitat quality. For a large research area, the traditional methods of supervised classification and manual visual interpretation are an inefficient use of time and labor [43]. Compared with the normal land-use/cover mapping method, Classification and Regression Trees (CART) is more suitable for analyzing and modeling complex land-use/cover data. This method automatically establishes the classification threshold and builds a decision tree based on manually selected training samples. It can comprehensively utilize the spectral information in the image and other auxiliary information to improve the classification accuracy [44,45,46].

The goal of this study is to evaluate the evolution of habitat quality and its influencing factors in the PLEEZ. First, we use the CART decision tree classification to map the land-use status of the PLEEZ from 1988 to 2018 and use the InVEST model to evaluate habitat quality. Second, we analyze the characteristics of changes in land-use and habitat quality. Finally, we evaluate the effect of the construction land expansion and forestland restoration on the quality of regional habitats in the PLEEZ. This study can provide guidance for urban development and environmental governance and protection in the study area.

## 2. Study Area, Materials, and Methods

### 2.1. Study Area

The Poyang Lake Eco-Economic Zone (PLEEZ) (27°30′ N–30°06′ N, 114°29′ E–117°42′ E) is a strategic plan proposed by the Jiangxi Provincial Party Committee and the Provincial Government of China in January 2008. At the end of 2009, the plan for the PLEEZ was approved by the Chinese government and improved the national development strategy of China. Its purpose is to scientifically and reasonably protect and develop Poyang Lake, making it a unique ecological zone with rapid economic development and coordinated urban and rural development.

**Figure 1:**
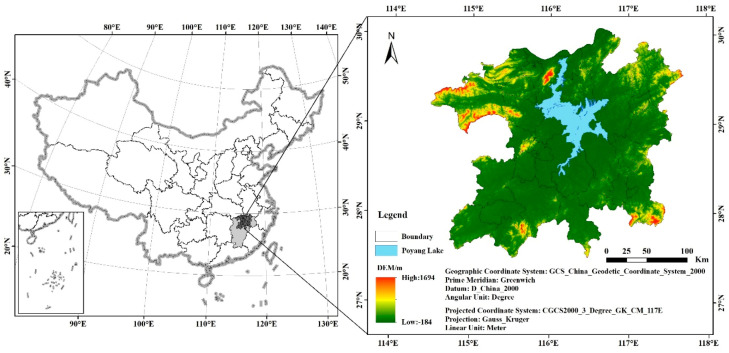
Location and topography of the Poyang Lake Eco-Economic Zone (PLEEZ).

The PLEEZ is located in northern Jiangxi Province, China, and includes the cities of Nanchang, Jiujiang, and Jingdezhen and parts of Yingtan, Xinyu, Fuzhou, Yichun, Shangrao, and Ji’an. The land area is approximately 5.12 × 104 km^2^, and the total population is approximately 20.006 million. It has a subtropical monsoon climate, with high temperatures and rain in summer, mild and humid winters, an annual rainfall of approximately 1500 mm, an average annual temperature of approximately 15 °C, and a frost-free period of approximately 260 days. The warm and humid climate and diverse soil types provide excellent conditions for the growth of forest vegetation (Figure 1). 

### 2.2. Landsat Imagery and Preprocessing

The remote sensing image data for this study are derived from the Landsat TM/OLI data with a spatial resolution of 30 meters from the U.S. Geological Survey (USGS) Earth Resources Observation and Science (EROS) Data Center. The PLEEZ is located within eight (120/40, 120/41, 121/39, 121/40, 121/41, 122/39, 122/40, and 122/41) WRS-2 paths/rows. Based on the growth period of the principal crops in the study area and comparing the crop growth images in different periods, the imaging time of the selected images was mainly between March and April. Due to the lack of data, the imaging time for some years was from June to December (Table 1).

The remote sensing image processing was as follows: First, ENVI5.3 software was utilized to perform radiometric calibration, atmospheric correction, cloudless image extraction, and image cropping on 28 Landsat TM/OLI scenes phase by phase. Second, the normalized vegetation index (NDVI) was calculated based on the following formula [47]. Third, Iterative Self-organizing Data Analysis (ISODATA) unsupervised classification was performed on the processed images with the maximum number of classifications as 10 categories and the number of iterations as 10. Finally, the blue, green, red, near-infrared, mid-infrared, and far-infrared bands included in the TM/OLI, the ISODATA unsupervised classification results, NDVI, and Digital Elevation Model (DEM) were merged into nine band images.

(1) $$NDVI=\frac{\rho_{NIR}-\rho_{red}}{\rho_{NIR}+\rho_{red}}$$

Classifying high spatial resolution aerial imagery using remote sensing analysis is an efficient way to get accurate land cover maps. The use of remote sensing techniques for land-use analysis in some areas has been examined in many studies [30,36,48,49]. The CART decision tree classification was utilized to classify the processed image. Compared with other multi-modeling methods, CART is simple and powerful. It is a relatively automatic machine learning method that requires relatively little data. It uses a guided algorithm to optimize the classification accuracy during the training process [46]. Therefore, it is suitable for analyzing and modeling complex land-use/cover data [44].

**Figure 2:**
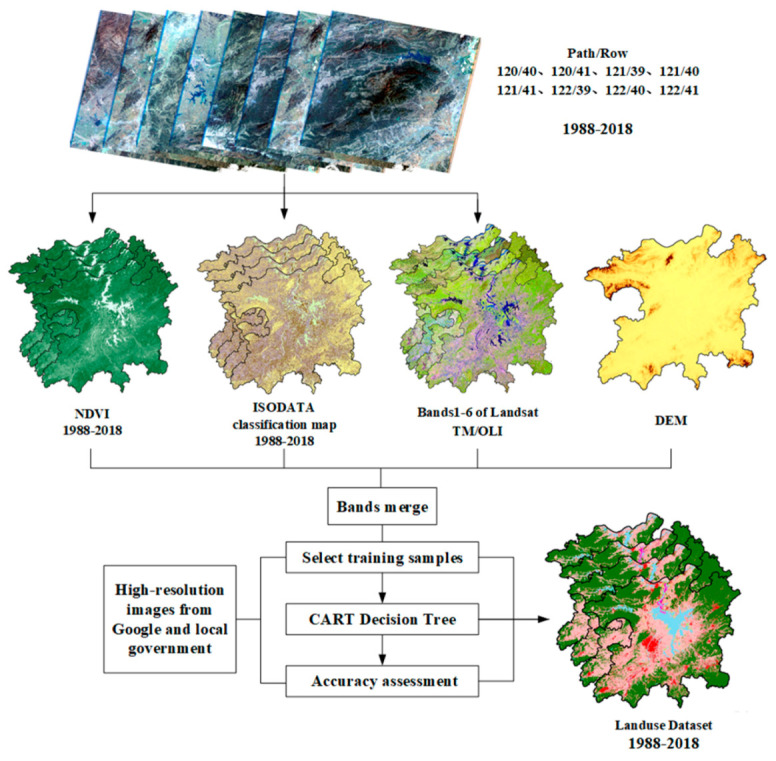
Land-use classification process.

The specific steps are as follows: First, ENVI5.3 software was used to select training samples for each image. Second, a classification decision tree was built and executed based on the selected training samples. Finally, the classification results for the remote sensing images from 1988 to 2018 were obtained, and then, their accuracy was evaluated. For the training sample selection and accuracy evaluation, we referred to high-resolution images of 1-m resolution obtained from Google Earth and local government departments (Figure 2).

The confusion matrix method was used to assess the accuracy of the land-use mapping results [50,51]. The accuracy assessment results show that the overall accuracies of the land-use data in the four stages were 84.83%, 86.26%, 93.47%, and 89.76% and that the kappa coefficients were 0.79, 0.81, 0.87, and 0.85, respectively. The accuracy was high, indicating that the classification results can be used for analysis of land-use and habitat quality changes (Table 2).

### 2.3. Habitat Quality Assessment

#### 2.3.1. InVEST Habitat Quality Evaluation Model

The Habitat Quality module in the InVEST model was used to evaluate the habitat quality. The basic principle of this method is to obtain the degree of habitat degradation by calculating the negative impact of threat factors on the habitat and then to calculate the habitat quality with the appropriate conditions of the habitat and the degree of degradation. The formula for calculating the degree of habitat degradation [30,52] is as follows:

(2) $$D_{xj}=\sum_{r=1}^{R}\sum_{y=1}^{Y_{r}}\left(\frac{\omega_{r}}{\sum_{r=1}^{R}\omega_{r}}\right)\times r_{y}\times i_{rxy}\times \beta_{x}\times S_{jr}$$

(3) $$i_{rxy}=\{\begin{array}{ll} 1-\frac{d_{xy}}{d_{r\max}} & \left(Linear\;decay\right)\\ \exp\left[-\left(\frac{2.99}{d_{r\max}}\right)\times d_{xy}\right] & \left(Exponential\;decay\right) \end{array}$$

where *D_xj_* is the degree of habitat degradation of grid *x* in land-use type *j*; *ω_r_* is the weight of threat factor *r*, which indicates the relative degree of damage to the habitat by the threat factor; *r_y_* is the intensity of threat factor *y*; *β_x_* is the resistance of the habitat to external interference; *S_jr_* is the relative sensitivity of the habitat to threat factors; *i_rxy_* is the influence of threat factor *r* in grid *y* on grid *x*; *R* is the total number of threat factors; *Y_r_* is the grid number of threat factor *r*; *d_xy_* is the distance between grid *x* and grid *y*; and *d*_*r*max_ is the maximum interference radius of threat factor *r*. The degree of habitat degradation is between 0 and 1. The larger the value, the higher the level of habitat degradation.

The habitat quality calculation formula [30,52] is as follows:

(4) $$Q_{xj}=H_{j}\times \left[1-\frac{D_{xj}^{z}}{D_{xj}^{z}+k^{2}}\right]$$

where *Q_xj_* is the habitat quality of grid *x* in land-use type *j*, *H_j_* is the habitat suitability of different land-use types, *k* is the half-saturation constant, and *z* is the scaling parameter. The habitat quality value is between 0 and 1. The higher the *Q_xj_* value, the better the habitat quality.

Based on the characteristics of the study area and the recommended values for the INVEST model determined through references [30,42,52] and expert consultations, cultivated land, construction land, and unused land were chosen as the threat factors. The parameters to be set for each threat factor are the maximum influence distance, weight, and decay type. In addition, the habitat suitability and sensitivity to threat factors for the various land-use types need to be established. The above parameters are shown in Table 3 and Table 4.

#### 2.3.2. Assess the Impact of Construction Land Expansion and Forestland Restoration on Habitat Quality

According to Song et al. [52] and McDonald et al. [53], we evaluated the impact of construction land expansion on habitat quality by calculating the habitat degradation rate caused by construction land expansion. The formula for evaluating the impact of construction land expansion on habitat quality is as follows:

(5) $$\Delta Q_{x}^{p_{1}-p_{2}}=\frac{Q_{x}^{\prime p_{2}}-Q_{x}^{p_{1}}}{Q_{x}^{p_{1}}}\times 100\%$$

where $\Delta Q_{x}^{p_{1}-p_{2}}$ is the change rate in habitat quality caused by construction land expansion in region *x* from *P*_1_ to *P*_2_, $Q_{x}^{p_{1}}$ is the habitat quality of region *x* in *P*_1_, and $Q_{x}^{\prime p_{2}}$ is the habitat quality in region *x* with the influence of construction land expansion between *P*_1_ and *P*_2_. The impact was calculated using the land-use data that updated the construction land expansion area of *P*_1_ to *P*_2_. However, the area without construction land expansion was not updated to avoid impact of areas beyond the construction land expansion. With the same method, we evaluated the effects of forestland restoration on habitat quality.

### 2.4. Hotspot Analysis of Habitat Quality Changes

Hotspot analysis (Getis-Ord Gi*) can be used to analyze whether the spatial variation in habitat quality shows high-value aggregation or low-value aggregation in local areas. With reference to the operation steps in Chen et al. [54], we completed a hotspot analysis in ArcGIS 10.3 and obtained the *p* and Z values. The *p* value represents the probability, and the Z value is a multiple of the standard deviation. When the *p* values are 0.01, 0.05, and 0.1, the confidence levels are 90%, 95%, and 99% and the corresponding Z values are <−1.65 or >1.65, <−1.96 or >1.96, and <−2.58 or >2.58, respectively. For positive z-scores with statistical significance, the higher the z-score, the higher the clustering of high values, indicating that the area is a concentration area with increased habitat quality (a hotspot). For negative z-scores with statistical significance, the lower the z-score, the tighter the clustering of low values, indicating that this area is a habitat quality loss concentration area (a cold spot).

## 3. Results

### 3.1. The Land-Use Mapping Results Based on CART

**Figure 3:**
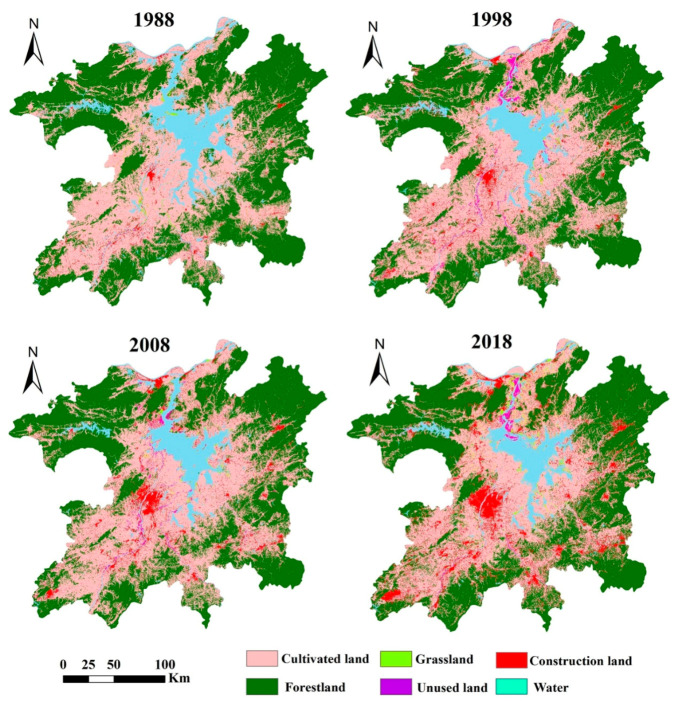
Spatial distribution of land use on the PLEEZ from1988 to 2018.

Figure 3 presents the results of CART decision tree classification mapping for the PLEEZ from 1988 to 2018. The land use in the study area showed obvious spatial differentiation characteristics. The forestland was widely distributed in the mountainous areas northwest and northeast of the study area. The water was mainly distributed in the central area, while the cultivated land and construction land were mainly distributed in the plain area between the mountains and Poyang Lake. Unused land and grassland were mainly distributed around rivers and lakes.

**Figure 4:**
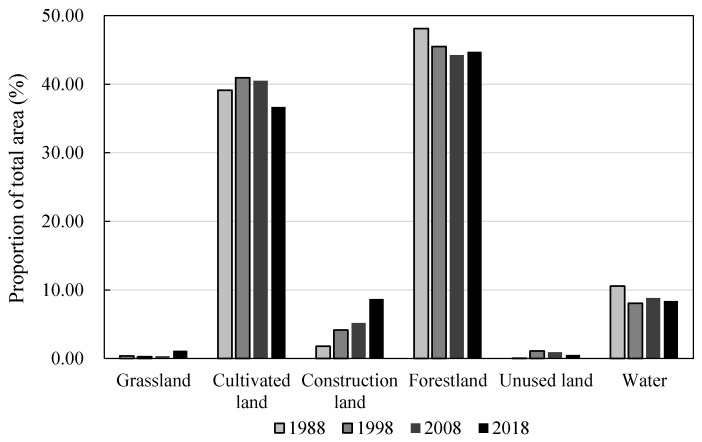
Land-use changes in the PLEEZ from 1988 to 2018.

From 1988 to 2018, land use in the PLEEZ changed significantly (Figure 4). Cultivated land and forestland were the dominant land-use types, accounting for more than 85% of the total area of the district. From 1988 to 2018, the cultivated land in the study area first increased slightly and then declined after 1998. The forestland gradually declined in the early stage and rebounded slightly in 2008–2018. There were few grasslands and unused lands, and they were mainly distributed in the tidal flats near lakes and rivers.

**Figure 5:**
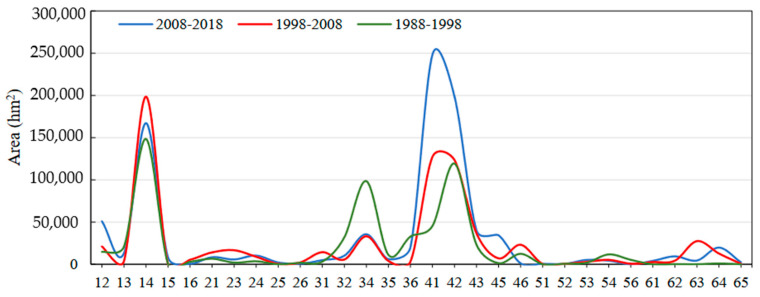
Changes in land-use types in the PLEEZ from 1988 to 2018. Note: Nos. 1–6 represent forestland, construction land, water area, cultivated land, grassland, and unused land, respectively. Code 12 represents forestland converted to construction land, and the other codes follow the same rule.

The land-use changes in the study area from 1988 to 2018 are shown in Figure 5. The most obvious land-use change typologies were “forestland-cultivated land” (code 14), “cultivated land-forestland” (code 41), and “cultivated land-construction land” (code 42).

From 1988 to 1998, the most obvious land-use change typology was “forestland-cultivated land” (code 14), followed by “water area-cultivated land” (code 34) and “cultivated land-construction land” (code 42), accounting for 24.72%, 16.39%, and 19.86% of the converted land-use types, respectively. From 1998 to 2008, the most obvious changes were “forestland-cultivated land” (code 14) followed by “cultivated land-forestland” (code 41) and “cultivated land-construction land” (code 42), which accounted for 28.16%, 18.06%, and 17.50% of the converted land-use types, respectively. From 2008 to 2018, the land-use types with the most obvious changes were “cultivated land-forestland" (code 41), “cultivated land-construction land” (code 42) and “forestland-cultivated land” (code 14), which respectively accounted for 27.12%, 21.69%, and 18.25% of the converted land-use types (Table 5).

The analysis shows that the principal land-use change typology in the study area from 1988 to 2008 was “forestland-cultivated land”. In 2008–2018, “cultivated land-forestland” and “cultivated land-construction” became the main types of land-use change.

### 3.2. Spatial Patterns of the Habitat Degradation Degree and Habitat Quality

#### 3.2.1. Habitat Degradation Degree

**Figure 6:**
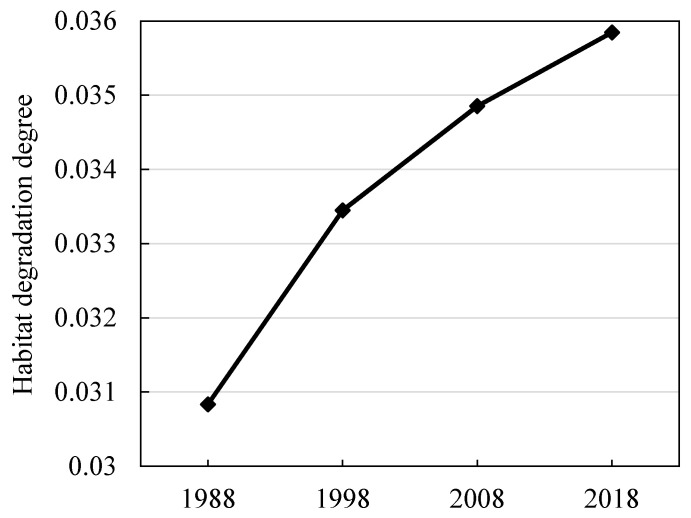
Habitat degradation degree in the PLEEZ from 1988 to 2018.

Figure 6 shows the average change in the degree of habitat degradation in the study area from 1988 to 2018. The results show that, during the period from 1988 to 2018, habitat degradation in the study area increased gradually. In the three periods of 1988–1998, 1998–2008, and 2008–2018, it increased by 8.47%, 4.20%, and 2.85%, respectively.

To better distinguish the characteristics of the variation in the degree of habitat degradation in different regions, we used NBC (natural break point classification) in ArcGIS to classify the degree of habitat degradation, which was divided into 5 levels: no degradation (0–0.025), mild degradation (0.025–0.05), moderate degradation (0.05–0.10), moderate-severe degradation (0.10–0.15) and severe degradation (≥0.15).

**Figure 7:**
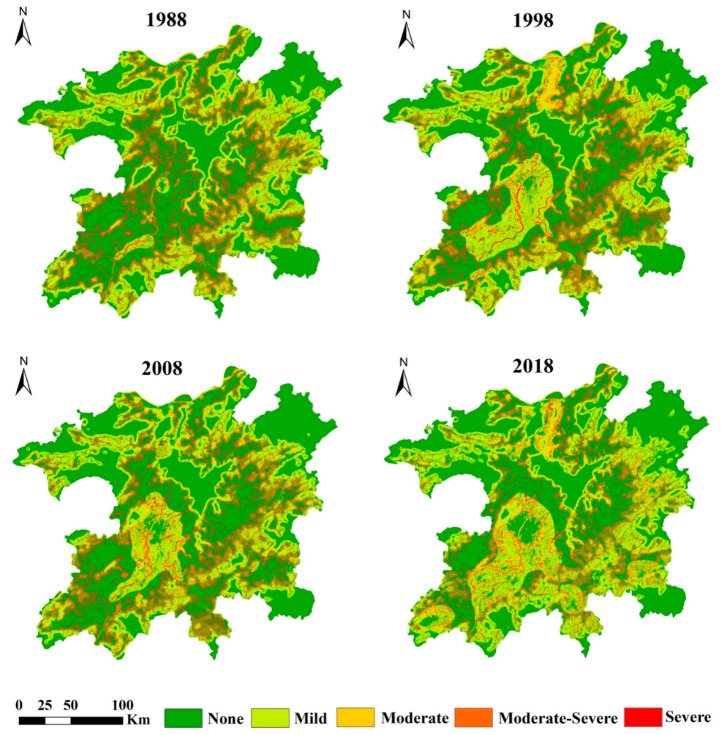
Spatial distribution of the habitat degradation levels in the PLEEZ from 1988–2018.

Spatial distribution of the habitat degradation levels is shown in Figure 7. The degradation level is severe, moderate-severe, or moderate and mainly distributed around the marginal areas of cities, mountains, rivers, and lakes, forming a ring structure. The land use changes frequently in these areas. Areas with degradation levels with no degradation or mild degradation were widely distributed in the inner areas of mountainous areas and large lakes. These areas have less human activity, and these land-use types are unlikely to change.

From 1988 to 2018, the area of habitat degradation in the study area continued to expand. The proportion of no degraded areas decreased significantly, while the proportion of mild and moderately degraded areas increased significantly (Table 6).

#### 3.2.2. Habitat Quality

**Figure 8:**
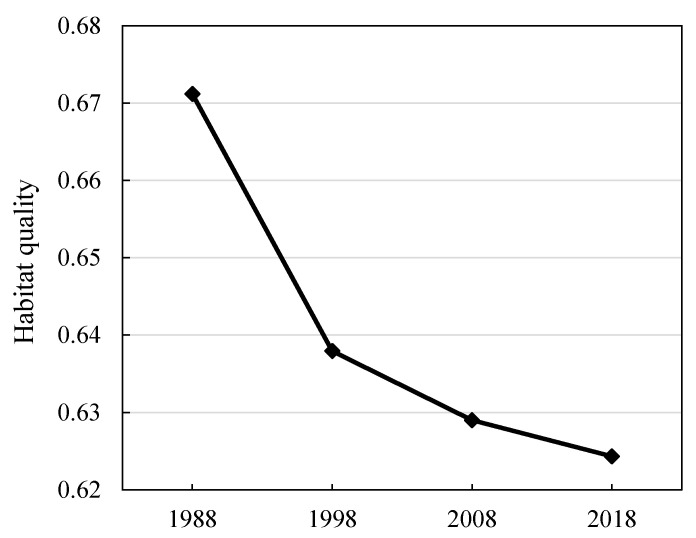
Habitat quality in the PLEEZ from 1988 to 2018.

Figure 8 shows the change in habitat quality in the study area. The results show that, from 1988 to 2018, the habitat quality in the study area decreased year by year. In 1988–1998, 1998–2008, and 2008–2018, the habitat quality decreased by 4.95%, 1.40%, and 0.74%, respectively. The habitat quality showed a declining trend, and the habitat quality change rate gradually decreased. This shows that the rate of habitat quality decline in the study area gradually slowed.

With reference to the classification standards in existing studies [55], the habitat quality of the study area was classified into five levels: very poor (0–0.2), poor (0.2–0.4), fair (0.4–0.6), good (0.6–0.8), and excellent (0.8–1.0).

**Figure 9:**
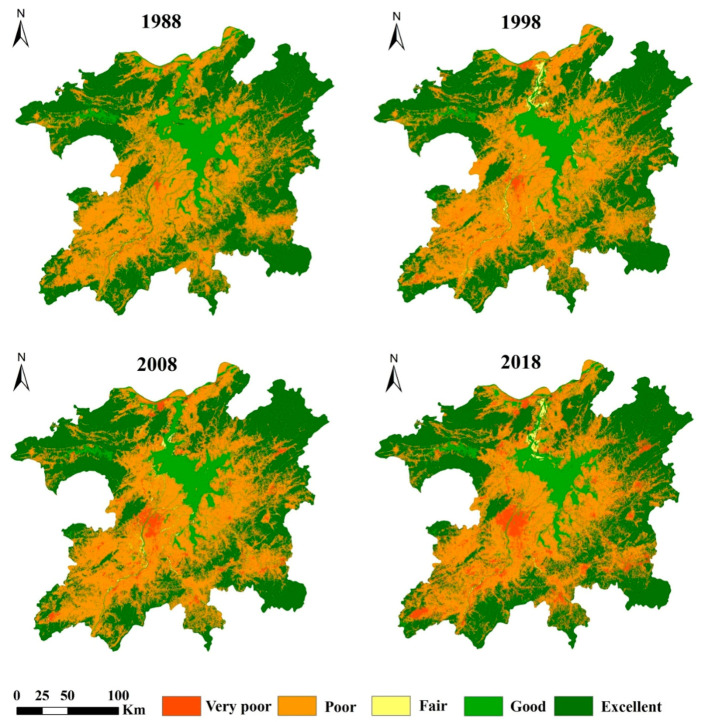
The spatial distribution of the habitat quality levels in the PLEEZ from 1988–2018.

Figure 9 shows that the areas with higher habitat quality are mostly located in the mountainous areas in the east, south, and northwest of the study area. The areas with low habitat quality are mainly distributed in urban built-up areas, especially in Nanchang City and other nearby cities and counties. During the period from 1988 to 2018, city expansion led to the gradual expansion of areas with lower habitat quality around the urban built-up area, encroaching the surrounding areas with higher habitat quality.

From 1988 to 2018, the areas with poor and very poor habitat quality grades in the study area accounted for 40.90%, 45.09%, 45.69%, and 45.35% of the area, respectively. The proportions of areas with excellent habitat quality were 48.47%, 45.77%, 44.57%, and 45.80%, respectively. The proportion of areas with very poor habitat quality increased year by year, from 1.79% in 1988 to 8.71% in 2018 (Table 7). This shows that the reduction in habitat quality in the study area has been suppressed but that the deterioration of habitat quality in regional areas is still intensifying. These deteriorated areas are mainly concentrated around newly added construction land near the city.

### 3.3. Effect of Land-Use Change on Regional Habitat Quality

**Figure 10:**
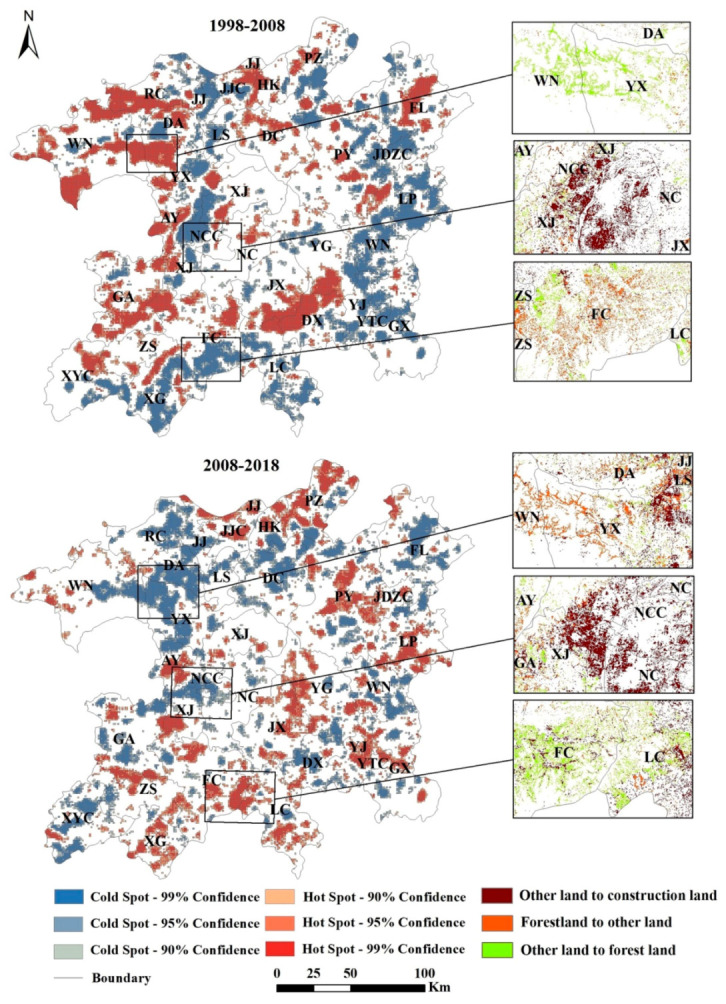
Cold spots and hotspots of habitat quality changes from 1998 to 2018 in the PLEEZ. Notes: The PLEEZ includes Pengze (PZ), Fuliang (FL), Hukou (HK), Jiujiang (JJ), Ruichang (RC), Jiujiang City (JJC), Poyang (PY), Duchang (DC), Wuning (WN), Dean (DA), Lushan (LS), Yongxiu (YX), Leping (LP), Xinjian (XJ), Yugan (YG), Anyi (AY), Nanchang (NC), Wannian (WN), Jinxian (JX), Gaoan (GA), Guixi (GX), Yujiang (YJ), Dongxiang (DX), Fengcheng (FC), Yingtan (YT), Linchuan (LC), Zhangshu (ZS), Xinyu (XY), Xingan (XG), Nanchang City (NCC), and Jingdezhen (JDZ).

Cold spots and hotspots with a confidence higher than 90% were graphed to show the cold spot and hotspot distributions with statistically significant clustering effects (Figure 10). The analysis shows that, from 1998 to 2018, the spatial distribution patterns of cold and hotspot habitat quality changes showed definite changes. In 1998–2008, the cold spot areas (where habitat quality declined) were mainly distributed in the rapid urbanization area along Poyang Lake and in the mountainous and hilly areas in the southeast. The hotspots (where habitat quality improvement) were mainly distributed in the mountainous and hilly areas in the middle and west of the study area, included Dongxiang, Gao’an, Anyi, etc. In 2008–2018, the cold spot distribution range decreased, mainly in the plains, especially near Nanchang City and Jiujiang City around Poyang Lake. The hotspot distribution became more scattered and is mainly distributed in the hilly areas southeast and northeast of the study area.

The main land-use changes in the cold spot area from 1998 to 2018 were the changes in forestland to other lands and from other lands to construction land. The principal land-use changes in hotspot areas were other lands to forestland (Figure 10). This result shows that construction land expansion and forestland degradation are the biggest threats to regional habitat quality while forestland restoration is the key to improvements in regional habitat quality.

## 4. Discussions

### 4.1. Interpretation of the Causes of Land-Use Change in the PLEEZ

The analysis in this paper shows that land-use changes in the PLEEZ are closely related to human activities and local government policies. The analysis shows that, from 1988 to 2018, forestland, cultivated land, and construction land were the most frequently converted land-use types in the PLEEZ.

From 1988 to 2008, “forestland-cultivated land” was the main land-use change typology, which was mainly attributed to human land development and deforestation activities [56,57]. Therefore, during this period, the forest area gradually decreased. In 2008–2018, “cultivated land-forestland” began to become the most important land-use change typology. This can be mainly attributed to the environmental protection plan for the PLEEZ, for example, the “One Big Four” project for afforestation and greening in the Poyang Lake Basin (beginning in 2008), the ecological construction project for soil and water conservation (beginning in 2010), the water resources ecological protection project for the source of the Yangtze River and Poyang Lake (beginning in 2011), and the Poyang Lake Basin Eco-environmental protection projects such as water conservation and forest construction and protection (beginning in 2011) [58]. These measures changed the trend in the area of forestland in the study area from decreasing to increasing.

From 1988 to 2008, the area of cultivated land did not change substantially and accounted for approximately 40% of the total area. This can be attributed to the comprehensive effects of land reclamation, construction sites, cultivated land abandonment, and other factors. However, after 2008, the area of cultivated land decreased significantly, which is primarily due to farmland being restored to forest and the encroachment of construction sites. The analysis in this article also confirmed this trend. During this period, “cultivated land-forestland” and “cultivated land-construction land” were the main land-use change typologies.

In addition, the conversions between water and other land-use types were also frequent from 1988–2018. This result is largely due to the changes in water quality and hydrological conditions of the Yangtze River [38,59]. Previous studies have implied that global climate change and human disturbance are the main reasons for the changes in hydrological conditions in the Poyang Lake area [60,61].

### 4.2. The Ecological Protection Measures in the PLEEZ Have Promoted Forestland Restoration, Which Is of Profound Significance for Improving Habitat Quality in the Poyang Lake Area

After establishment of the PLEEZ, vegetation coverage has increased, vegetation degradation has reduced, and the obvious changes in the regional land-use types are mainly toward cultivated land and forestland. Forestland protection has been the main reason for the change in vegetation cover trends [62,63]; this finding reflects the research results in this article.

The analysis in this paper shows that, from 1998 to 2008, the most obvious land-use change typology in the study area was “forestland-cultivated land”. The area converted from forestland to cultivated land accounted for 28.16% of the total land-use change area. “Cultivated land-forestland” and “cultivated land-construction land” were also notable changes in this period. Forestland degradation and construction land expansion lead to habitat quality degradation. After establishing the PLEEZ in 2008, the conversion of forestland to cultivated land decreased from 28.16% in 1998–2008 to 18.25% in 2008–2018. At the same time, the main land-use change typology at this stage was “cultivated land-forestland”, which accounted for 27.12% of the total land change area. This shows that the ecological protection project has achieved good results and that the overall forest area has also improved after continuous decline.

**Figure 11:**
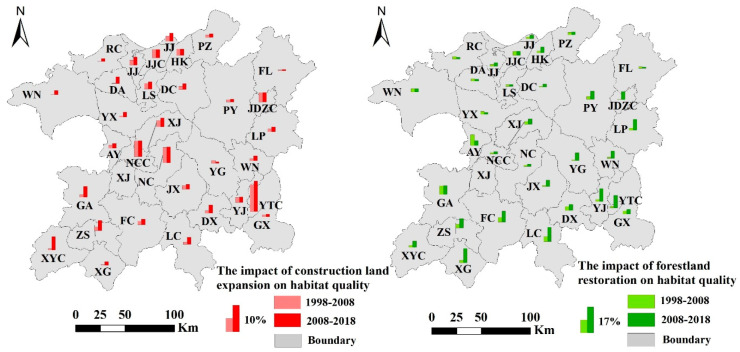
The impact of construction land expansion and forestland restoration on habitat quality. Note: please refer to Figure 10.

According to Equation (5), unambiguous information about the changes in habitat quality caused by forestland restoration in 1998–2008 and 2008–2018 was obtained (Figure 11). In 1998–2008 and 2008–2018, the area of cultivated land that was restored to forest in the study area (cultivated land-forestland) increased from 1351.76 km^2^ to 2818.59 km^2^. The proportions of regional habitat quality improvement due to forestland restoration were 2.94% and 6.19%, respectively. This shows that ecological protection projects have increasing effects on habitat quality improvement, so they can further suppress the rate of degradation of the local habitat quality.

From the perspective of spatial distribution, the influence of forestland restoration on habitat quality is mainly distributed in the surrounding mountainous and hilly areas. The implementation of returning farmlands to forests project has obviously restored forests and grassland in these areas [62]. In the future, we should strengthen and consolidate the achievements of ecological restoration in these areas. At the same time, other counties that are rich in forests should also be regarded as the key ecological conservation sites, especially the cities and counties located around the Lushan Mountains and the Jiuling Mountains. These areas are rich in forests and play a key role in regional ecological security.

### 4.3. Despite the Positive Significance of Ecological Protection Projects for Habitat Protection, Excessive Urbanization Is Still the Greatest Threat to Habitat Quality

The analysis in this paper shows that the construction land in the PLEEZ expanded from 1988 to 2018 and caused serious degradation of habitat quality. According to Equation (5), this study provides unambiguous information about habitat quality losses caused by construction land expansion in 1998–2008 and 2008–2018 (Figure 11). In 1998–2008 and 2008–2018, the average annual growth rate of construction land in the study area increased from 2.44% to 6.77%. The proportion of regional habitat quality decline owing to the expansion of construction land, being 1.92% and 3.74%, respectively. Acceleration of the growth of construction land in the region makes the impact of construction land expansion on habitat quality more serious.

Figure 11 shows that the large-scale expansion of large cities such as Nanchang, Jiujiang, and Yingtan will seriously threaten the quality of surrounding habitats. Therefore, we suggest that the large-scale and disorganized expansion of the central cities of the PLEEZ should be limited in the future to reduce the impacts of urban expansion on habitat quality.

### 4.4. Policy Implications and Future Directions

**Figure 12:**
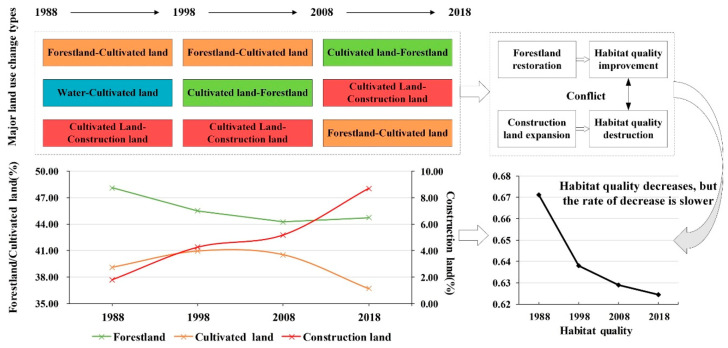
The logical framework of habitat quality changes in the PLEEZ.

These results show that regional land-use and habitat quality in the PLEEZ changed significantly. The long-term situation of forest degradation is reversed, and the area of forestland increased, which contributed to regional habitat quality improvement. However, at the same time, urbanization continues to accelerate, which reduces habitat quality. Therefore, under the influence of these two aspects, although the habitat quality of the PLEEZ is still declining, the downward trend has gradually slowed (Figure 12).

“Ecological protection” and “economic development” are often in conflict, but the ecological and environmental conditions are extremely relevant to human well-being and sustainable development [54]. China’s rapid economic growth and urban expansion have come at the cost of resource depletion and environmental degradation [64,65]. Land-use planners are facing the challenge of striking a balance between food production, economic growth, and natural resource protection [39]. Given these contradictory goals, China’s current land use policies and plans are ineffective in responding to rapid urbanization, in protecting cultivated land and natural resources, and in protecting the environment [64,65,66].

The report of the 19th National Congress of the Communist Party of China in 2017 emphasized ecosystem protection and the prohibition of large-scale development of the Yangtze River Economic Belt [54]. To promote green development and high-quality development of the Yangtze River Economic Belt, the “Yangtze River Protection Law” is also in the process of approval. To ensure sustainable and coordinated environmental and socioeconomic development under rapid urbanization, in the future, it is necessary to prevent forest degradation and water pollution caused by human activities and to curb the continued decline in habitat quality. In mountainous areas, the policy of “returning farmland to forest” should continue to be implemented, as it can effectively restore the declining habitat, maintain biodiversity, and provide ecosystem balance. At the same time, it is necessary to improve the land-use efficiency of urban built-up areas and to avoid disorganized expansion of large cities to prevent construction land expansion from further threatening habitat quality. Additionally, urban greening can be considered to offset the negative impact of urban development [67]. The green GDP can be considered in the performance evaluation of government officials, and a comprehensive performance evaluation system for population, resources, environment, economy, and society can be established, which will help to fundamentally ensure the implementation of sustainable development strategies.

Previous studies on habitat quality mostly focused on a single perspective, such as the impact of urbanization development on habitat quality [30,31,52]. Compared with these studies, this study takes the PLEEZ as the study area and analyzes the impact of land-use changes on regional habitat quality from the perspectives of urbanization development and ecological protection. It can provide a reference for formulating habitat protection policies in a similar region. In addition, we used CART decision tree classification to quickly and accurately map land-use change over a large area by improving on previous research. Compared with research using 1000 × 1000 m data [55] and research through supervised classification and human–machine interactive interpretation methods [30,52], faster and more accurate land-use information can be obtained. 

Changes in habitat quality and land-use are influenced by a set of complex processes of natural factors (e.g., temperature and precipitation) and human disturbances (e.g., dams, weirs, and levees) [36]; the attribution of these effects will be discussed in future studies. In addition, it is important to understand that the habitat suitability of natural forestland is likely higher than that of planted forestland. However, in this study, due to the limitations of image resolution and data acquisition, planted forestland and natural forestland were not considered separately. In the future, it may be appropriate to use more accurate land-use data to improve this study.

## 5. Conclusions

The focus of this study was on analyzing the temporal and spatial evolution of land use and habitat quality in the PLEEZ. This article discussed the effect of land-use change on habitat quality in detail. The main conclusions are as follows:

Land-use changes dominated habitat quality variations in the PLEEZ. Natural conditions are the fundamental determinant of the spatial distribution of habitat quality, and human activities are the main factors influencing habitat quality changes. Due to the natural geographical conditions, areas with better habitat quality in the study area were mostly distributed in mountainous areas and most of the poorer-quality areas were distributed in urban built-up areas in the plains. 

Ecological protection measures can improve regional habitat quality, while excessive urbanization is a threat to habitat quality. After 2008, establishing the PLEEZ accelerated the progress of urbanization and caused serious damage to the habitat quality of the study area. However, at the same time, the implementation of ecological protection projects such as returning farmland to forest slowed a decline in habitat quality. Under the influence of these two factors, the habitat quality of the PLEEZ gradually declined but the rate of decline was suppressed.

This study discovered the trends in habitat quality changes in the PLEEZ and provided a reference for formulating habitat protection policies in the region and other similar research areas. 

## Figures and Tables

**Table 1:** Summary of the Landsat images used in this study.

Year		Imaging Time			
		2018	2008	1998	1988
paths/rows	120/40	2018/4/19	2008/3/14	1998/5/30	1988/7/5
	120/41	2018/4/19	2008/3/14	1998/12/8	1988/7/5
	121/39	2018/4/10	2009/3/16	1995/12/7	1989/7/15
	121/40	2018/3/9 2018/4/10	2009/3/16	2000/5/10 1996/12/9 1999/12/10	1989/6/13 1989/7/15
	121/41	2018/3/9	2009/3/16	1999/12/10	1989/7/15
	122/39	2018/4/17	2009/4/8	1998/7/15	1987/9/19
	122/40	2018/4/17	2009/4/8	1999/12/17	1988/7/3
	122/41	2018/4/17	2009/4/8	1995/12/14	1988/7/3

**Table 2:** Accuracy assessment results from 1988 to 2018.

Year	Overall Accuracy	Kappa Coefficient
1988	84.83	0.79
1998	86.26	0.81
2008	93.47	0.87
2018	89.76	0.85

**Table 3:** Threat factors and their characteristic parameters.

Threat Factor	The Maximum Influence Distance/km	Weights	Decay Type
Cultivated land	4	0.6	linear decay
Construction land	8	0.4	exponential decay
Unused land	6	0.5	linear decay

**Table 4:** Sensitivity of each land-use type to habitat threat factors.

Land-Use Type	Habitat Suitability	Threat Factor		
		Cultivated Land	Construction Land	Unused Land
Cultivated land	0.3	0.0	0.8	0.4
Forestland	1.0	0.6	0.4	0.2
Grassland	1.0	0.8	0.6	0.6
Water	0.7	0.5	0.4	0.2
Construction land	0.0	0.0	0.0	0.1
Unused land	0.6	0.6	0.4	0.0

**Table 5:** Amount of land-use changes in the PLEEZ from 1988 to 2018.

	1988–1998		1998–2008		2008–2018	
	Area (hm^2^)	Change Ratio (%)	Area (hm^2^)	Change Ratio (%)	Area (hm^2^)	Change Ratio (%)
12	14,600.25	2.43	21,035.88	2.98	50,878.89	5.56
13	20,936.34	3.49	3761.19	0.53	13,016.52	1.42
14	148,410.27	24.72	198,507.24	28.16	167,158.08	18.25
15	579.87	0.10	349.74	0.05	8006.22	0.87
16	3029.58	0.50	5324.67	0.76	557.46	0.06
21	6510.51	1.08	14,041.98	1.99	8349.12	0.91
23	1909.8	0.32	16,577.46	2.35	5689.8	0.62
24	3456.27	0.58	8841.69	1.25	10,209.55	1.11
25	101.88	0.02	403.83	0.06	2105.46	0.23
26	1726.83	0.29	2198.97	0.31	631.89	0.07
31	3096.63	0.52	14,276.25	2.02	4879.71	0.53
32	31,889.34	5.31	5579.46	0.79	10,114.47	1.10
34	98,396.91	16.39	33,113.7	4.70	35,520.48	3.88
35	11,168.37	1.86	3898.8	0.55	6088.23	0.66
36	32,733.99	5.45	3179.79	0.45	20,236.77	2.21
41	45,490.77	7.58	127,326.6	18.06	248,375.43	27.12
42	119,243.16	19.86	123,345.99	17.50	198,647.91	21.69
43	23,070.42	3.84	36,474.48	5.17	39,844.8	4.35
45	1270.8	0.21	6998.13	0.99	34,265.25	3.74
46	12,350.7	2.06	23,053.5	3.27	1129.14	0.12
51	93.24	0.02	329.31	0.05	505.35	0.06
52	566.1	0.09	503.73	0.07	475.92	0.05
53	1889.1	0.31	2815.83	0.40	5084.91	0.56
54	11,631.6	1.94	5163.39	0.73	4187.79	0.46
56	5231.16	0.87	683.82	0.10	66.51	0.01
61	14.4	0.00	2520.81	0.36	4064.31	0.44
62	149.76	0.02	4232.88	0.60	9337.41	1.02
63	42.03	0.01	27,381.24	3.88	4402.35	0.48
64	877.05	0.15	12,694.14	1.80	19,722.87	2.15
65	0.00	0.00	412.74	0.06	2211.57	0.24
Total	600,467.13	100.00	705,027.24	100.00	915,764.17	100.00

Note: please refer to Figure 5.

**Table 6:** The proportion of different habitat degradation levels in the PLEEZ from 1988–2018.

Habitat Quality Levels	Proportion of Different Habitat Degradation Level (%)			
	1988	1998	2008	2018
None	64.49	59.68	59.37	57.54
Mild	14.50	18.70	18.04	22.10
Moderate	10.37	10.71	10.78	11.91
Moderate-severe	6.90	6.84	7.79	6.21
Severe	3.73	4.07	4.01	2.25
Total	100	100	100	100

**Table 7:** The proportion of different habitat quality levels in the PLEEZ from 1988–2018.

Habitat Quality Levels	Proportion of Different Habitat Quality Level (%)			
	1988	1998	2008	2018
Very poor	1.79	4.25	5.17	8.71
Poor	39.11	40.84	40.52	36.64
Fair	0.05	1.10	0.91	0.43
Good	10.58	8.04	8.83	8.42
Excellent	48.47	45.77	44.57	45.80
Total	100	100	100	100
